# To test or not to test: what we have learnt from 50 years of dynamic testing in acromegaly

**DOI:** 10.1007/s11102-026-01649-x

**Published:** 2026-03-31

**Authors:** Laura De Marinis, Sabrina Chiloiro, Antonio Mancini, Penelope Giambò, Antonella Giampietro, Antonio Bianchi, Alfredo Pontecorvi, Andrea Giustina

**Affiliations:** 1 Dipartimento Di Medicina e Chirurgia traslazionale, Facoltà Di Medicina e Chirurgia, Università Cattolica Del Sacro Cuore, Rome, Italy; 2 Division of Endocrinology, Metabolic Disease and Internal Medicine, Fondazione Policlinico Universitario A. Gemelli IRCCS, Milan, Italy; 3 Institute of Endocrine and Metabolic Sciences, San Raffaele Vita-Salute University and IRCCSSan Raffaele Hospital, Milan, Italy; 4 Fondazione Policlinico Gemelli, IRCCS, Università Cattolica Del Sacro Cuore, Rome, Italy

**Keywords:** Oral glucose tolerance test, Acromegaly, Somatotroph, Growth hormone, Insulin like growth factor i

## Abstract

Acromegaly is a rare pituitary disorder characterized by the inappropriate secretion of growth hormone (GH) by a pituitary adenoma in most cases. Diagnostic criteria of acromegaly have significantly changed over the last 25 years, providing different cut-offs of serum GH levels both in basal conditions and after glucose inhibition. Historically, due to several analytical limitations in GH and insulin-like growth factor I (IGF-I) assays, the confirmation of clinically suspected acromegaly was primarily based on dynamic tests involving the administration of substances known to acutely and physiologically modulate GH secretion. In some cases, paradoxical GH responses have been observed during these dynamic tests. The oral glucose tolerance test (OGTT), performed with the administration of 75 g of glucose, is currently the only dynamic test recommended by clinical guidelines in acromegaly. Initially, a paradoxical response during OGTT was defined as the failure of GH suppression following glucose exposure. More recently, this definition has evolved with the introduction of additional criteria, including the percentage increase in GH levels, the GH ratio, and temporal parameters. Over time, the GH response during OGTT has been used for different purposes. Initially, it served as a diagnostic tool; however, the most recent consensus statements recognize its role as a prognostic indicator of long-term remission following neurosurgical treatment. Therefore, considering the predictive value of certain dynamic tests in assessing treatment response in acromegaly, this review aims to provide a comprehensive overview of the evolution of dynamic testing in acromegaly, with a specific focus on tests associated with paradoxical GH responses.

## Introduction

Acromegaly is a rare pituitary disorder characterized by the inappropriate secretion of growth hormone (GH) by a pituitary adenoma in most cases, with consequent increase in liver production of insulin-like growth factor-I (IGF-I) under GH hyperstimulation [1].

Acromegaly is characterized by increased morbidity and mortality, especially in patients with long term exposure to high IGF-I levels [2, 3] due to acromegaly-related comorbidities, such as cardiovascular and metabolic disorders and cancer [4–6].

In acromegaly, the achievement of an early disease control is strictly correlated to a better prognosis and reduced incidence of acromegaly-related comorbidities [7, 8]. Therefore, reducing the still existing diagnostic delay to achieve disease control as soon as possible (in the natural history of the disease) is the main goal in the management of acromegaly [1].

Diagnostic criteria of acromegaly have significantly changed over the last 25 years, providing different cut-offs of serum GH levels both in basal conditions both after glucose inhibition [4–11]. In last expert consensus on acromegaly diagnosis, random GH assessment was recognized not useful for diagnostic purpose, due to the physiological pulsatile secretion of GH by pituitary somatotroph cells [12]. Furthermore, GH rhythmicity may be preserved in some patients with acromegaly, along with the specific differences in secretion related to sex and age [13]. Variations in GH sensitivity due to GHR polymorphisms may explain the heterogeneous IGF-I responses observed in acromegalic patients, even at similar levels of GH secretion [14].

Historically, the only way for endocrinologists to confirm the clinical suspicion of acromegaly was through the use of a familiar diagnostic approach with dynamic testing [15], which involved the administration of substances known to physiologically and acutely influence GH secretion [12]. The need to perform dynamic tests was due to several analytical issues in GH and IGF-I dosage. The radioimmunoassay for determining GH levels became available about 60 years ago, despite both intrinsic and pre-assay variability due to pulsatile GH secretion and the influence of many endocrine and metabolic factors persist. Moreover, reliable methods to assay IGF-I and neuroradiological techniques were not available for the subsequent two/three decades [12].

Interestingly, in some cases, during dynamic testing, GH paradoxical response can be observed: it can be defined as the opposite findings than those observed in non-acromegaly patients, for example, an increase rather than a decrease or no response of GH during “dynamic” testing [16].

Besides giving important pathophysiological insights into the GH neuro-regulation in acromegaly [17], some of these dynamic tests [such as the oral glucose tolerance test] proved to be very useful for diagnostic and follow-up purposes [18] while others opened the way for novel therapeutic approaches, such as the dopamine agonist [16]. Moreover, the outcome of these tests anticipated the great heterogeneity among acromegalic patients, which was demonstrated many years later in terms of different tumor characteristics [19].

With the advent of reliable IGF-1 assay and of advanced neuroradiological techniques, most of the dynamic tests were progressively abandoned for the diagnosis and follow-up of acromegaly, also due to both the dramatic decrease in hospital budgets and to the unavailability of testing substances in the market. For these reasons and for the recent renewed clinical interest in the predictive value of dynamic tests on response to treatment in acromegaly, we aimed in this mini review to perform a detailed reappraisal of the evolution of the role of dynamic testing in acromegaly, specifically addressing the tests in which paradoxically GH responses were observed.

## Dopamine

Among neurotransmitters, particular attention was given to dopamine (DA), which has a stimulatory effect on GH secretion in healthy individuals. Indeed, its precursor, the L-DOPA (L-3,4-dihydroxyphenylalanine) was extensively used to evaluate GH deficiency both in children and adults. The effect of L-DOPA can be enhanced by β-blockers pretreatment.

In acromegaly, by contrast, dopamine exerts a suppressive effect, mainly due to the binding to the subtype 2 of the dopamine (D2) receptors on tumor cells, as proved by “in vivo” study [20, 21]. Successively, it has also been demonstrated that dopamine receptors can dimerize with subtype 5 somatostatin receptors [22, 23].

The comparative effects of different dopaminergic agents in acromegaly have also been investigated, showing a greater reduction in GH levels after administration of the DA, which does not cross the blood-brain barrier (BBB) – compared with L-dopa or bromocriptine, both of which do cross the BBB [24].

Interestingly, in the study of Hanew and his collaborators, when L-dopa was administered 20 min after the start of domperidone infusion, GH levels increased, resulting in a paradoxical response. The authors therefore hypothesized a dual effect of the L-dopa: a direct suppressive action on the pituitary somatotroph cells and an indirect stimulatory effect via the hypothalamus. Consequently, the inhibition or stimulation may represent the net outcome of these different pathways [24].

In our experience, a paradoxical response to L-dopa was present in about 65% of patients with acromegaly, occurring more frequently in small adenomas [class I of Hardy classification] and in patients with lower basal GH levels [25]. Conversely, as expected, antidopaminergic drugs may stimulate the GH secretion in acromegaly [26]. Indeed, sulpiride was shown to elicit a GH response in a subgroup of patients, such as domperidone, which does not cross the blood-brain barrier, suggesting a direct effect at the infundibular/pituitary level [27].

Studies on paradoxical response to dopamine represented the pathophysiological basis for the clinical use of dopaminergic agonists, which were historically the first effective medical therapy in acromegaly [28]. In fact, initially bromocriptine [28] and successively the more tolerated and convenient cabergoline, either alone [29] or in combination with somatostatin receptor ligands (SRLs) [30], have become therapeutic options in acromegaly. Currently, almost 50 years after the discovery of the paradoxical GH response to dopamine in acromegaly, cabergoline is still used as an off-label drug [31] and is recommended by consensus guidelines [32] in the treatment of mild acromegaly, being an oral and inexpensive option [1].

## Thyrotropin releasing hormone [TRH] and other neuropeptides

In addition to dopamine, several other neuropeptides have also been used in dynamic testing in acromegaly, which did not appear to elicit any relevant GH response in acromegaly [33]. Conversely, a paradoxical acute GH response has been observed after the administration of the luteinizing hormone-releasing hormone (LHRH), which physiologically only stimulates pituitary release of gonadotrophins [34].

### TRH

Among neuropeptides, the most studied has been thyrotropin-releasing hormone (TRH), which physiologically stimulates thyrotropin and prolactin. In fact, a paradoxical GH response to TRH was first described in 1972 [34, 35] in 50–75% of untreated patients [36, 37] and was not influenced by increased cortisol levels [38]. However, this response was not specific, as it has also been demonstrated in anorexia nervosa [39], type 1 diabetes mellitus [40], primary hypothyroidism [41] and severe liver and kidney failure [42, 43].

The mechanism of action of TRH was extensively debated, but a conclusive explanation was not found. In fact, a direct effect on tumoral cells at receptor or post-receptor level, a paracrine mechanism, a disturbance of hypothalamic-pituitary connections, the co-secretion of GH and prolactin in pituitary adenomas or the inhibition of somatostatin release by TRH itself were hypothesized but not proven over the years [44]. Notably, TRH test is more likely to yield a positive response in small pituitary tumors rather than in larger ones [45]. Another hypothesis could be related to the common differentiation pathway of thyrotrophs and somatomammotrophs, both characterized by the expression of Pit-1, according to the most recent pituitary adenoma classification [46].

Although not unequivocally [44], most studies suggested a predictive role of the paradoxical GH response to TRH on the success of pituitary surgery [37, 47, 48]. Interestingly, in a cohort of 50 patients, we found that a pre-operative paradoxical response to TRH (seen in 33 out of 50 patients, 66%) was correlated with smaller tumor volume [49] and predictive of curative surgery [45].

Besides its predictive value on surgical remission, the paradoxical GH response to TRH was not able to predict either the responsiveness to long-term dopaminergic or SRLs treatment [50, 51] or differentiate patients bearing the gsp mutation [52]. Therefore, pending its availability, TRH test could be hypothesized to still have a potential role in assessing early post-surgical remission since IGF-1 may need several months to normalize [53], or in patients with acromegaly and type 2 diabetes in whom glucose tolerance test (OGTT) is contraindicated [5] or in patients with suspected post-surgical hypothyroidism which is not always easy to diagnose [54, 55]. However, routine applications of TRH testing remain largely theoretical because of the need of a pre-surgery testing, and as potentially serious side effects were seldom reported after TRH administration in patients with macroadenomas [56], as most patients at acromegaly diagnosis [1, 57].

### Galanin

More recently, among other neuropeptides, galanin has been shown to determine a paradoxical GH inhibition in patients with acromegaly [58] likely exerting this potent effect directly on GH-secreting adenoma cells [59, 60]. In fact, galanin stimulates GH secretion in normal subjects in a similar but synergistic way to GH-releasing hormone (GHRH) [61], particularly in young females [62]. Variable GH decrease occurred after galanin infusion in 90% of patients with active acromegaly, correlating with the rate of GH increase after TRH [63]. Whereas a physiological GH stimulatory effect was observed in patients with acromegaly, undergoing surgical remission [63]. Galanin was shown to exert its paradoxical inhibitory effect also in acromegaly patients with type 2 diabetes without significant acute effects on blood glucose, suggesting that it could be used in place of OGTT for confirming diagnosis and remission of acromegaly, in patients with hyperglycaemia when OGTT is contraindicated [64] (Table 1).

**Table 1:** GH Paradoxical response to L-DOPA/dopamine, TRH, LHRH, and Galanin tests, definition and correlation with acromegaly outcome. Abbreviation: L-DOPA: L-3,4-dihydroxyphenylalanine; TRH: Thyrotropin-releasing hormone; LHRH: luteinizing hormone-Releasing hormone

Authors, year (Ref.)	Definition of paradoxical response	Patients (number)	Correlation between GH paradoxical response and outcome
De Marinis et al., 1993 [25]	General decrease in GH levels after administration of L-Dopa	34	Smaller tumor volume and lower basal GH levels.
Biermasz et al., 2002 [37]	Absolute GH increase of 3.75 mU/l after TRH administration	129	Higher risk of disease recurrence.
Chin et al., 2013 [44]	TRH ratio (the peak/basal ratio of GH) > 2 after TRH administration	41	No correlation with tumor volume.
De Marinis et al., 2002 [45]	Serum GH increase greater than 50% of basal values after TRH administration	50	Inversely related to the tumor size.
Faglia et al., 1978 [47]	General increase in GH levels after administration of TRH	18	Absence of paradoxical GH response to TRH indicates satisfactory treatment of acromegaly.
Brockmeier et al., 1993 [48]	General increase in GH levels after administration of TRH and LHRH	20	No correlation with recurrence after surgery.
Giustina et al., 1995 [64]	General decrease in GH levels after administration of Galanin	23	GH decrease after galanin infusion occurred in patients with active acromegaly. A physiological GH increase after galanin infusion was observed in patient with surgical remission of acromegaly.
Mazziotti et al., 2008 [65]	General decrease in GH levels after administration of Galanin	17	Galanin infusion can be used to confirm diagnosis or remission of acromegaly in patients affected by type 2 diabetes, in place of OGTT.

## Oral glucose tolerance test

### Insufficient Inhibition

Nowadays, the only dynamic test recommended by guidelines in acromegaly is the OGTT, which is performed by administering 75 g of glucose orally and measuring serum GH levels at baseline and every 30 min for up to 2 h [9]. However, indications and interpretations varied significantly over time. In fact, whereas in the first consensus on acromegaly (that was released 25 years ago), OGTT testing for GH dosage was mandatory both in diagnosis and follow-up [9], in more recent consensus, its indications were circumscribed to patients in whom post-surgery remission could not be univocally determined based on baseline GH and IGF-I levels [10, 11, 66]. Moreover, the nadir level of GH above which the fall in GH can be deemed insufficient differentiating physiological and acromegaly response progressively varied with the advent of ultrasensitive assays from 1 ng/ml in first consensus to 0.4 ng/ml more recently. Also, BMI thresholds for interpretation were proposed [66].

Interestingly, the use of OGTT in clinical practice for confirming diagnosis and remission of acromegaly is not based on a paradoxical response but on an insufficient GH decrease as compared to physiological thresholds of serum GH observed after acute blood glucose in non-acromegaly subjects [12].

Several pathophysiological mechanisms have been proposed to explain the insufficient fall below the physiological thresholds of GH levels after acutely increased of blood glucose. According to some authors, this reduced GH secretion could be attributable to increased somatostatin production by hypothalamic cells induced by hyperglycaemia [65, 67]. According to other authors, the lower secretion of GH after oral glucose load would be due to a reduced somatotroph sensitivity to GHRH-induced by hyperglycaemia [68, 69]. More recent theories focused on the role of insulin in determining suppression of GH secretion. In fact, the rise in insulin production induced by hyperglycaemia, given its high concentration, allows insulin itself to bind insulin receptors on somatotroph cells, mainly inhibiting the release of GH already synthesized from cytoplasmic vesicles [70]. Furthermore, this insulin role would occur primarily during the sporadic insulinemic peaks that follow hyperglycaemic phases, rather than under conditions of chronic hyperinsulinism, such as those found in individuals with insulin resistance [71]. In these settings, in fact, it is possible that chronic hyperinsulinism could rather lead to an increase in somatostatin tone.

### Paradoxical increase

Interestingly, in about one-third of patients with acromegaly, it is well known that a paradoxical GH response to OGTT may be observed, characterized by an increase in GH values, rather than by a simple insufficient inhibition [72, 73]. However, since relevant spontaneous fluctuations of GH levels may also occur in patients with acromegaly [74, 75], a critical point is to reach a consistent and universally accepted definition of GH paradoxical response, above which the GH increase may be reasonably attributed to a glucose-dependent GH stimulating effect, based also on current ultrasensitive assays of GH.

In the last decade, a renewed interest in a better definition of the paradoxical GH increase during OGTT was observed, to define the still unresolved questions on epidemiology, pathophysiology and clinical implications of the GH paradoxical response. According to different authors, the GH paradoxical response has been defined with very variably either as percent increase in GH versus baseline or as GH ratio (between peak and baseline value) ranging from 20% [76, 77], to 25 [78] or 30% [79]. More recently, a temporal criterion was also introduced in the definition using the OGTT ratio with peak occurring within the 90 min point [80, 81] and being at least 1 ng/ml with an absolute increase of at least 0.6 ng/ml versus baseline [80].

Table 2 summarizes the articles that discuss GH paradoxical response during OGTT.

**Table 2:** GH paradoxical response to oral glucose tolerance test (OGTT), definition and correlation with acromegaly outcome. Abbreviation: GH ratio: OGTT ratio is calculated as the ratio between the peak GH value during OGTT and the baseline value; fg-SRLs: first generation somatostatin receptor ligand; SRL: somatostatin receptor ligand, GIP: Glucose-dependent Insulinotropic Polypeptide; GIPR: Glucose-dependent insulinotropic polypeptide receptor. Table 2A: The paradoxical response was defined as a general increase of GH after glucose administration. Table 2B: The paradoxical response was defined as percentage of increase of GH after glucose administration. Table 2C: The paradoxical response was defined as an increased in the GH ratio after glucose administration

Authors, year (Ref.)	Definition of paradoxical response	Patients (number)	Correlation between GH paradoxical response at OGTT test and outcome
A			
Umahara et al., 2003 [82]	Increase in GH	4	GIP could be responsible for the paradoxical response, either through binding its receptor on the transformed somatotrophs cells, or through the presence of an alteration in the GIP mediated pathway in regulating GH secretion.
Occhi et al., 2011 [83]	Increase in GH	21	GIPR overexpression
Hage et al., 2019 [84]	Increase of GH	38	GIPR overexpression GNAS wild-type
B			
Atquet et al., 2021 [79]	GH levels > 25% respect to pretest basal GH level	30	Advanced age, smaller tumors, higher IGF-I levels at the diagnosis Better response to SRLs
Mukai et al., 2019 [80]	GH levels > 30% respect to pretest basal GH level	64	Higher IGF-I levels at the diagnosis Higher glucose and insulin levels at 120 min Better response to treatment with dopamine agonist and fg-SRLs
Jensen et al., 2025 [85]	GH levels > 30% respect to pretest basal GH level	25	Nearly 30% of acromegaly patients can exhibit a paradoxical GH increase during OGTT
Scaroni et al., 2019 [77]	GH ratio >120%	496	Elderly patients Higher GH levels/tumor volumes ratio with a slower growth and less invasive tumors Better response to fg-SRLs
	OGTT GH ratio > 120%	198	Good response to surgical and medical therapies and to overall treatments Association with the activation of GIP/GIPR pathway.
Ceccato et al., 2024 [81]	OGTT ratio ≥ 120% at least 90 min after glucose load in association with a GH zenit > 1 µg/L and with an absolute increase of at least 0.6 µg/L	60	Milder tumor phenotype and good response to medical therapy Higher prevalence of diabetes and higher risk of developing diabetes during Pasireotide treatmentHigher glucose peak during OGTT
Losa et al., 2025 [86]	GH ratio ≥120% at latest 90 minutes after glucose load	254	Older patients, with smaller tumors and less invasive growth Lower risk of disease recurrence after surgical remissionGood response to first generation SRLs administration
Occhi et al.,2025 [87]	GH ratio ≥120% achieved within 90 min after glucose load	59	Higher probability of glucose alterationNo difference in worsening of glucose metabolism during Pasireotide administration

The main pathophysiological mechanism underlying the paradoxical GH response to OGTT was hypothesized to be the involvement of the pathway glucose-dependent insulinotropic polypeptide (GIP) and GIP receptor (GIPR) belonging to the G protein coupled receptors. The activation of this pathway would be able to both stimulate GH secretion and exert a trophic effect on somatotroph cells, similarly to GHRH [86]. Indeed, the binding of GHRH to its G protein–coupled receptor increases intracellular cAMP, which is essential for the proper function of somatotroph cells [82].

Interestingly, already 30 years ago, a non-uniform GH response to OGTT in acromegaly, which was not present after intravenous glucose administration, was reported, suggesting a role of peripheral somatostatin release and of GIPR in mediating the paradoxical GH response [72]. Similarly, subsequently, it was reported that a patient with acromegaly exhibited an increase in GH levels after oral but not intravenous glucose administration, also suggesting that GIP could be involved in the paradoxical response, likely through binding its receptor, expressed on tumoral somatotrophs [86] as confirmed by subsequent cellular and molecular studies [83, 88].

A very recent study confirmed that nearly 30% of acromegaly patients can exhibit a paradoxical GH increase during OGTT (> 30% vs. baseline), most commonly within the first 90 min [84]. Notably, in this study, around 50% of patients with paradoxical response showed a reduction in GH levels under GIPR blockade in association with a more pronounced expression of GIPR in tumoral cells obtained after surgery, supporting the importance of GIP/GIPR pathways [no changes in patients with insufficient inhibition]. However, elevated expression of GIPR was also found among some patients belonging to the non-paradoxical response group, without differences in age, sex, adenoma volume or cavernous sinus invasion. On the other hand, higher prolactin levels at diagnosis were found among the non-paradoxical group. Therefore, GIP could sustain the paradoxical response in patients, carrying tumors with strong expression of GIPR, suggesting that GIPR antagonist may represent a future therapeutic option [85].

Importantly, other studies reported some relevant molecular and clinical implications also in terms of outcome prediction for patients showing GH paradoxical response during OGTT. In fact, pathologically they seemed to harbour adenomas with a higher level of copy-number alteration and higher expression of GIPR [in adenomas negative for GNAS mutations] [83, 88]. Furthermore, higher fasting and post-prandial GIP levels were observed in acromegaly patients compared to healthy subjects [89]. Moreover, concerning treatment outcome prediction, the patients with paradoxical response to OGTT were reported to be older, carrying smaller, slowly growing and less cavernous sinus invasiveness adenomas, higher IGF-1 levels, lower frequency of hyperprolactinemia and with greater chances of disease remission after surgery [77] and medical therapy with SRLs [76–79] and bromocriptine [79].

Finally, as far as glucose metabolism is concerned in acromegaly, patients with the paradoxical response had higher blood glucose and insulin levels at 120 min of OGTT than the other acromegaly patients. Moreover, in these patients, diabetes was found more frequently, often associated with a deficit of first-phase insulin secretion [77—80]. In consideration of the metabolic alterations most commonly present in these patients, caution was suggested [90] in using a second-generation analogue, such as pasireotide [87]. Importantly, due to the current wide use of incretins for the treatment of obesity, a note of caution in the use of GIP analogs in acromegaly seems reasonable [87] at least in patients with paradoxical GH increase after glucose, according to the potential stongest GIPR expression in somatotroph adenomas.

Table 2 summarizes the articles that discuss GH paradoxical response during OGTT.

In the last decades we learnt from translational and clinical studies that acromegaly is an heterogeneous disease with often unpredictable outcomes due to many factors including the variability in biological characteristics of the GH-secreting pituitary adenomas [1]. In fact, the pathological [91, 92] and neuroradiological [93] characterization of the adenoma are now considered predictors of outcomes and useful to personalize the treatments [94, 95]. Interestingly, despite the huge progress in those two areas, the accuracy on the identification of the best treatment and outcome is still limited, waiting for further contribution of artificial intelligence [96]. Interestingly, in contrast to the pathological and neuroradiology fields, no advancements have been made in the biochemical characterization and assessment of the disease over the last few decades. Conversely, there is a shift toward diagnostic simplification, with a diminishing role for baseline and stimulated GH secretion tests. Instead, IGF-1 is becoming the primary reference parameter for both biochemical diagnosis and long-term follow-up [66]. While this simplified approach facilitates diagnosis—particularly in non-specialist settings [1, 97] and enables the monitoring of all therapeutic interventions, including pegvisomant [98], it has notable limitations. From a biological perspective, IGF-1 is an indirect marker of adenoma activity rather than a reflection of its intrinsic biological features; furthermore, clinical assessment is complicated by significant variability among commercial assays. These issues are well underlined by the frequent discrepancy in clinical practice of the GH and IGF-1 levels when measured together [14].

In a recent survey of pituitary centers of excellence, the oral glucose tolerance test (OGTT) was the only dynamic test consistently performed across all centers [99]. Rather than being viewed as obsolete, the OGTT provides critical insights into the complex and heterogeneous pathophysiology of the disease, with diagnostic and prognostic purposes. Indeed, the latest consensus recognizes that a GH nadir below 1 ng/mL (or 0.4 ng/mL with modern ultrasensitive assays) during an OGTT performed three months post-neurosurgery is a significant prognostic indicator of long-term remission [87].

Consistent with previous findings, recent data suggest that while a paradoxical GH response to the OGTT is rare, it may predict a favorable response to adenoma-targeted treatments [87], despite its association with a higher risk of developing diabetes. Considerable uncertainty persists regarding the precise definition of a paradoxical GH increase. Although the most recent consensus defines it as a GH rise of more than 20% over baseline, alternative cut-offs have been proposed. Consequently, further studies are warranted to establish a standardized definition, which would significantly enhance the clinical utility of this test.

Given its simplicity, cost-effectiveness, and established diagnostic and prognostic value, it is reasonable to advocate for the expanded use of the OGTT in all patients with acromegaly both before and shortly after surgery, including those managed outside of specialized centers. For patients with diabetes, where the OGTT is contraindicated, other provocative tests like TRH or galanin may serve as alternatives in expert centers [100], especially when surgical cure is unlikely. Clinicians should exercise caution with the TRH test in patients with large adenomas and must account for the limited availability of these pharmacological agents.

In conclusion, dynamic GH evaluation remains a cornerstone of the diagnostic and prognostic framework for acromegaly. Beyond providing relevant insights into the complex and heterogeneous pathophysiology of the disease, these tests have significant therapeutic implications.

Therefore, their use should not be abandoned; rather, expertise in these protocols should be maintained within Pituitary Tumor Centers of Excellence (PTCOEs). In these specialized settings, comprehensive biochemical data are indispensable for a personalized therapeutic approach, particularly for difficult cases, ultimately leading to optimized disease outcomes. 

## Data Availability

No datasets were generated or analysed during the current study.
